# Defining and evaluating novel procedures for involving patients in Core Outcome Set research: creating a meaningful long list of candidate outcome domains

**DOI:** 10.1186/s40900-018-0091-5

**Published:** 2018-03-02

**Authors:** Harriet Smith, Adele Horobin, Kathryn Fackrell, Veronica Colley, Brian Thacker, Deborah A. Hall, for the Core Outcome Measures in Tinnitus (COMiT) initiative

**Affiliations:** 1 National Institute for Health Research (NIHR) Nottingham Biomedical Research Centre, Ropewalk House, 113 The Ropewalk, Nottingham, NG1 5DU UK; 2 Otology and Hearing Group, Division of Clinical Neuroscience, School of Medicine, University of Nottingham, Nottingham, NG7 2UH UK; 3 Nottingham University Hospitals NHS Trust, Queens Medical Centre, Derby Road, Nottingham, NG7 2UH UK

**Keywords:** Tinnitus, Outcome domains, Delphi survey, Public research partners, Reducing, Consolidating, Modifying

## Abstract

**Plain English summary:**

Outcome domains are aspects of a condition that matter to patients and clinicians and can be measured to assess treatment effects. For tinnitus, examples include ‘tinnitus loudness’ and ‘ability to concentrate’. This study focuses on the first stage of agreeing which outcome domains should be measured in all clinical trials of tinnitus. Crucially, it involves identifying outcome domains, prior to a voting process. This article describes how we effectively involved patients in that study design process, and reflects on the impact of their input.

The study first compiled a long list of all possible outcome domains before asking interested parties, including patients, to vote which ones to include. Ensuring patients fully participate in this process holds unique challenges as it can be long, repetitive and its purpose far removed from their needs. These challenges may be addressed by involving patients in designing the research. There is evidence that other research teams are doing this, but its reporting is not detailed enough to guide others. Our paper seeks to address this.

We describe how we involved patients (people living with tinnitus) in creating a long list of outcome domains that we included in our study. We also reflect on the benefits this brought. Two patients partnered with us in designing the survey. We also consulted an independent patient review panel. Involving patients reduced the list of domains included in the survey and made domain names and associated descriptions clearer. Our resulting survey performed well in recruiting and retaining patients as participants.

**Abstract:**

**Background** Tinnitus is a complex audiological condition affecting many different domains of everyday life. Clinical trials of tinnitus interventions measure and report those outcome domains inconsistently and this hinders direct comparison between study findings. To address this problem, an ongoing project is developing a Core Outcome Set; an agreed list of outcome domains to be measured and reported in all future trials. Part of this project uses a consensus methodology (‘Delphi’ survey), whereby all relevant stakeholders identify important and critical outcome domains from a long list of candidates. This article addresses a gap in the patient involvement literature by describing and reflecting on our involvement of patients to create a meaningful long list of candidate outcome domains.

**Methods** Two Public Research Partners with lived experience of tinnitus reviewed an initial list of 124 outcome domains over two face-to-face workshops. With the Study Management Team, they interpreted each candidate outcome domain and generated a plain language description. Following this, the domain names and descriptions underwent an additional lay review by 14 patients and 5 clinical experts, via an online survey platform.

**Results** Insights gained from the workshops and survey feedback prompted substantial, unforeseen modifications to the long list. These included the reduction of the number of outcome domains (from 124 to 66) via the exclusion of broad concepts and consolidation of equivalent domains or domains outside the scope of the study. Reviewers also applied their lived experience of tinnitus to bring clarity and relevance to domain names and plain language descriptions. Four impacts on the Delphi survey were observed: recruitment exceeded the target by 171%, there were equivalent numbers of patient and professional participants (*n* = 358 and *n* = 312, respectively), feedback was mostly positive, and retention was high (87%).

**Conclusions** Patient involvement was an integral and transformative step of the study design process. Patient involvement was impactful because the online Delphi survey was successful in recruiting and retaining participants, and there were many comments about a positive participatory experience. Seven general methodological features are highlighted which fit with general principles of good patient involvement. These can benefit other Core Outcome Set developers.

**Electronic supplementary material:**

The online version of this article (10.1186/s40900-018-0091-5) contains supplementary material, which is available to authorized users.

## Background

### The core outcome measures in tinnitus (COMIT) initiative

This methodology article is based on experience involving the public in the COMIT’ID study. COMIT’ID stands for ‘Core Outcome Measures in Tinnitus – International Delphi’ and used a modified Delphi survey to seek consensus between different stakeholder groups about what outcome domains are critically important when deciding if an intervention for tinnitus is working. The COMIT (Core Outcome Measures in Tinnitus) initiative is led by members of the European TINnitus NETwork (TINNET) to develop standards for clinical trials in tinnitus [1, 2]. A core outcome set establishes standards for outcome selection and reporting in clinical trials of interventions to improve the wellbeing of people with this lifelong condition. An outcome can be viewed in two parts. First, the outcome domain refers to what aspects of tinnitus matter to patients and clinicians. Outcome domains refer to any aspect of tinnitus that is or can be experienced by a patient, such as ability to concentrate, sense of control, or impact on work. Second, the outcome instrument refers to how that domain is to be measured. Throughout this report, the term “outcome” refers to the general construct which includes both concepts of what and how to measure, while the term “outcome domain” or “domain” is restricted to the concept of what to measure. The list of critically important outcome domains - known as a Core Outcome Set (COS) - forms a minimum set of outcome domains that should be measured and reported in every clinical trial [3, 4]. A minimum set means that researchers must report on the same core outcome domains, whilst remaining free to collect and explore other outcomes too. COMIT’ID aims to develop three separate COSs for tinnitus: one for sound-based intervention outcome domains; one for psychology-based intervention outcome domains; and one for pharmacology-based intervention outcome domains. These COSs will be relevant for adults with chronic subjective tinnitus that should be measured and reported in every clinical trial of these interventions [5]. The long-term goal is to standardise what is measured when sound-based, psychology-based, or pharmacology-based tinnitus interventions are tested, so that data can be compared or combined across studies.

There are different ways that patients can be involved in the process of designing, running and disseminating a COS study. While the term “patient” typically refers to a person who is receiving or registered to receive medical intervention, “patient” is used here to refer to anyone who has lived experience of tinnitus. This article documents a set of methods for involving patients in creating the long list of tinnitus-related outcome domains for round 1 of the Delphi survey. These methods form the first stage of the consensus process to create the three COSs. The article also reflects on the impact of that particular involvement. Patients who took this role are referred to throughout as ‘Public Research Partners’, although others may use terms such as ‘advocates’, ‘representatives’, ‘contributors’, ‘surrogates’ or ‘community stakeholders’ etc. [6, 7].

### Why are core outcome sets (COS) needed for tinnitus?

Tinnitus is a subjective condition for which patients experience a diversity of complaints. Hence, there is no straightforward outcome. Some tinnitus outcomes are specific (for example, the perceived pitch of the tinnitus) while others are broad (for example, tinnitus-related quality of life). One of the challenges is that different studies evaluating interventions for tinnitus often measure and report different outcomes [8]. This makes it very difficult to compare results between studies. What is urgently needed are specific discussions around the major therapeutic approaches for tinnitus (namely sound therapies, psychological interventions, and pharmacological therapies) because they do not necessarily target the same tinnitus-related complaints [2, 5]. For example, if all sound-based intervention studies reported the same outcomes, then all the results could be compared and combined. A methodological consensus regarding what aspects of tinnitus should be measured would help to make sense of all the knowledge produced and thus improve the rate of progress in developing interventions [2]. In addition, involving patients in developing these outcome reporting standards would go a long way to ensuring its relevance to end users [2, 5].

### Defining candidate outcome domains

Defining a COS involves working with various stakeholders, such as health care users, health care practitioners and commercial representatives, to prioritise large numbers of outcome domains and achieve consensus about the minimum set. Various methods have been used to identify important outcomes when developing a health care COS and there is insufficient evidence to determine which is the most appropriate or efficient [9]. Delphi survey methods are one of the most frequent approaches used [9]. In a conventional Delphi survey, participants nominate outcomes in round 1 to be considered in subsequent rounds, but a modified Delphi survey is becoming a popular way to reduce the burden to participants [9, 10]. In a modified Delphi survey, a comprehensive list – called a ‘long list’ – of all possible outcomes is first identified through a scoping process such as a systematic review of previous clinical trials, and/or patient-centred responses collected through interviews or focus groups. This long list forms the first of several rounds in which participants rate the importance of each and every outcome [9, 11]. As part of round 1, it is generally recommended that participants be given opportunities to suggest new outcomes which they think are missing and submit feedback or personal perspectives about specific outcomes [3, 9, 10]. This enables the team to be confident that they are representing the perspectives of all relevant stakeholder groups and to gain a greater understanding of why a particular outcome might be deemed important.

### Procedures for involving patients in COS development

From a series of discussion-based workshops with patients, Young and Bagley [7] reported on how including patients as participants and as Public Research Partners holds unique challenges. Workshop delegates advised that Public Research Partners have an important role in developing clear explanations of COS and associated concepts because these can seem far removed from the experience of patients. Delegates also cautioned that the long list of candidate outcome domains found in some Delphi surveys can be off putting for many patient participants, and so Public Research Partners can again play an important role in highlighting this risk and helping to work out ways to minimise its occurrence. If not adequately addressed at the study design stage, these challenges could have a negative impact on accessibility of the Delphi survey to patients. Indeed, low rates of recruitment have been reported in a previous hearing-related COS [12, 13]. Increasingly, taking a patient-centred approach can improve the relevance and interest to patients. There is recent evidence that investigators are incorporating public involvement in a variety of different stages of COS development. Examples include appointing Public Research Partners to the Project Steering Group [14], reviewing and reducing the long list [14, 15], reviewing and suggesting new outcomes for the long list [16], deciding the outcome name [17], creating a plain language description for each outcome domain to ensure all participants interpret concepts as intended [18, 19], creating age-appropriate outcome domain descriptions with children and parents [20], creating ‘lay equivalents’ for a public version of the survey, different from the professional version [21], and piloting the Delphi survey for accessibility [16, 18, 22–24].

Despite these positive advances in COS methodology, Young and Bagley [7] found that published reports did not always clearly distinguish between public participation and involvement. Furthermore, while published protocols broadly state which steps of the process the public are to be involved, they generally do not provide any in-depth detail on the procedures to be used and the anticipated benefits to be gained [16–21, 23]. As such, Gargon and colleagues [25] conclude that there is a need for greater methodological guidance in how to effectively involve the public in COS development, while Jones and colleagues [26] have called for further research about the impact of these forms of public involvement. An illustrative example of good reflective practice is shown by Bruce and colleagues [13]. Their COS study presented plain language descriptions of outcome domains for the public participants only, not the professional stakeholders. However, the consensus meeting identified some domain names which had been ambiguous in meaning to the professional Delphi participants indicating that in future studies the plain language descriptions should be available to all.

### Aim

When designing the COMIT’ID study, no published procedures were found which described exactly how Public Research Partners have been involved in creating a long list of candidate outcome domains for a round 1 Delphi survey, nor evaluation of its impact. This methodology article addresses this gap in knowledge by reporting in detail our procedures to involve two Public Research Partners. The article also reflects on and evaluates the benefits of this form of public involvement. In so doing, the evidence contributes to the sharing of good practice in public involvement in developing COS. To further enhance the quality, transparency, and consistency of the PPI evidence base for public involvement in core outcome set development, reporting is guided by the GRIPP2-SF checklist [27].

## Methods

The Study Management Team comprised three tinnitus researchers (HS, KF, DAH). Public involvement was delivered by a dedicated team of two Public Research Partners with lived experience of tinnitus (VC, BT) and a Patient and Public Involvement and Engagement Manager (PPI/E Manager, AH). All the Study Management Team was also part of the wider COMIT’ID Research Steering Group that included three physicians from France, Germany, and Portugal. Independent lay reviews were provided by 14 members of the British Tinnitus Association’s Users’ panel, again all with lived experience of tinnitus, and five clinical experts who were members of the British Tinnitus Association Professional Advisory Committee. To improve the appeal to healthcare users and to reduce attrition, the two Public Research Partners additionally commented on the feasibility of the Delphi survey design, and reviewed study documentation (advertisements, Information Sheets, video instructions for the survey). Because these procedures for public involvement are not the focus of the current article, they are not reported further. All planned public involvement activities were described in the project protocol (version 2, dated 13 March 2017) which was approved by the West Midlands - Solihull Research Ethics Committee (reference 17/WM/0095) on 21 March 2017.

**Fig. 1:**
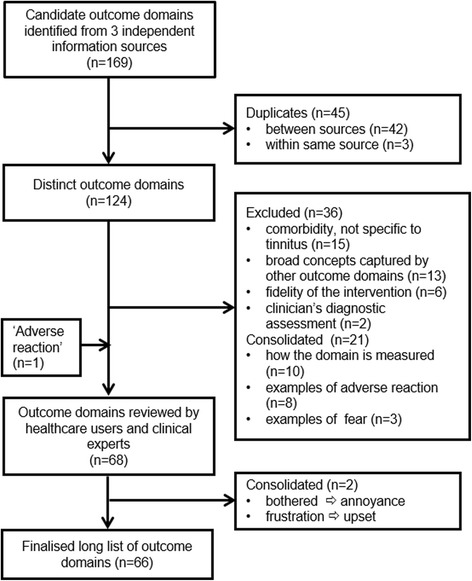
Flow diagram illustrating the pre-Delphi stage that was completed with the involvement of healthcare users as Public Research Partners prior to round 1 of the modified Delphi survey. Taken from Fackrell et al. [5]

The Study Management Team first sought to create a comprehensive list of all possible outcome domains relevant to tinnitus, regardless of intervention type. A common long list of outcome domains was therefore created for round 1 of all three Delphi surveys (sound, psychology and drug-based interventions). The researchers first identified 169 potential candidate outcome domains via: i) a systematic review of outcome domains reported in clinical trials of tinnitus interventions in adults (62 outcome domains) [8], ii) a thematic analysis of the items taken from 23 commonly reported patient-reported questionnaire instruments for tinnitus (64 outcome domains) [Fackrell, personal communication], and iii) a systematic review of dimensions of tinnitus-related complaints reported by patients and their significant others using questionnaire- and interview-based methods (43 outcome domains) [28]. The Study Management Team were able to reduce this list to 124 candidate outcome domains by consolidating domains that were literal duplicates of one another (see Fig. 1). Two members of the Study Management Team (HS, KF) and our two Public Research Partners (VC, BT) and PPI/E Manager (AH) then participated in two half-day workshops, with the initial objective to review the list of domains identified by the scoping exercise and their associated plain language descriptions; specifically to comment on the readability of the domain descriptions and on the suitability of the headings under which outcome domains were grouped. The team was motivated by the need to describe domains so that all participants, including patients, interpreted the meanings clearly and consistently across stakeholder groups. Also, that having English as an additional language would not act as a barrier to comprehension, given the international focus of the COMIT’ID study. Discussion focussed on the outcome domains themselves, not on their potential relevance to any sound-based, psychology-based, or pharmacology-based intervention.

In preparation for these workshops, and to create a starting point for discussion, the Study Management Team compiled preliminary plain language descriptions taken from the systematic reviews and content analysis, and from searches of the Oxford English Dictionary [29]. The Public Research Partners were encouraged to explain what each outcome domain name meant to them and then suggest a way to describe it using language that would be accessible to other members of the public. A harmonised plain language description had to have approval from both Public Research Partners and the researchers, before moving the discussion to the next domain. Although domains were presented one by one, there was cross-referencing to previously discussed domains where needed. Each workshop was audio recorded for later reference. Handwritten notes to aid discussion were encouraged and the two researchers wrote down the plain language description as well as any strong opinions or concerns voiced by either Public Research Partner. Such insights led to a number of unforeseen decisions about the long list of outcome domains for the round 1 Delphi survey.

The public involvement work with the two Public Research Partners was then supplemented with independent reviews conducted by 14 members of the public with tinnitus recruited from the British Tinnitus Association Users’ Panel. These reviewers responded to an invitation emailed to 30 members of the panel. Reviewers were naïve to the details of the COMIT’ID study, but were nevertheless experienced Lay Reviewers. Here, outcome domain names, plain language descriptions and category labels that had been developed for each domain in the modified long list were presented to Lay Reviewers using an online survey platform (Survey Monkey), with the option for free text responses about each domain. For each plain language description, Lay Reviewers were asked to select whether it was “a clear and understandable definition (no changes needed)” or not. If not, then they were asked to explain why and suggest alternative words or phrasing. Lay Reviewers were also asked to review the outcome domain category labels. For each outcome domain, Lay Reviewers were asked to select whether it “fit in this category” or not. If not, then they were asked to explain why and suggest an alternative category.

This yielded further insights which informed the final version of the long list for round 1 of the Delphi survey.

## Results

The 124 outcome domains from the scoping process were presented to the Public Research Partners and PPI/E Manager for generating plain language descriptions, and these were then reviewed by expert Lay Reviewers. The initial impression of the Public Research Partners was that the original long list was so long it was not user friendly, and their concerns were that this would negatively impact on recruitment and on sustained respondent engagement across the three survey rounds. This insight prompted the Study Management Team to critically rethink the composition of the long list leading to decisions to modify it in a number of radical, but unforeseen, ways. Illustrative examples of changes made to the long list of candidate outcome domains as a direct result of public involvement are reported below.

### Reducing the long list

The most notable unforeseen impact of the public involvement insights was to shorten the initial long list. It was reduced by 57 outcome domains leaving a final long list of 66 distinct, tinnitus-related domains. Overall, 36 domains were excluded, and 21 were pooled (i.e. consolidated) (see Fig. 1) either through direct recommendation by the Public Research Partners, or through Study Management Team discussion following the workshops.

The input of the two Public Research Partners had the greatest impact on the final content of the long list. Subsequent comments made by the Lay Reviewers led to only two further reductions in the number of candidate domains (see Fig. 1).

#### Excluding broad concepts captured by other outcome domain concepts

The Public Research Partners argued that 13 outcome domains were broad concepts whose meaning was already encapsulated by other domains in the long list (Fig. 1). In one example, the general domain ‘cognitive difficulties’ was deemed to be covered by the more specific domains: ‘concentration’, ‘tinnitus-related thoughts’, ‘confusion’, and ‘ability to ignore’. For example, on ‘tinnitus-related thoughts’, BT said “*Thoughts about your tinnitus…Thoughts about tinnitus...’cause that basically is the cognitions*”. In a second example, ‘health-related quality of life’ was deemed to be part of ‘impact on relationships’, ‘impact on individual activities’, ‘impact on social life’, ‘impact on work’, and ‘sexual difficulties’.

Such overlap in meanings was considered to risk causing confusion when rating the importance of individual outcome domains in the modified Delphi survey. To minimise this risk, the Public Research Partners recommended that such broad concepts should be removed from the long list and instead used as a category label for grouping similar domains together for presentation in the modified Delphi survey.

#### Consolidating equivalent outcome domains

The Public Research Partners considered that three fear-related concepts (‘Fear for health’, ‘Fear for quality of life’ and ‘Fear of tinnitus becoming worse’) could not easily be distinguished from one another. For example, the discussion of the domain ‘fear for quality of life’, cross-referenced back to the domain ‘fear for health’. BT said “*That’s so close to that one. It’s the same question asked in a different way.*” and VC said “*When you look at it later, you’ll be able to see if it’s turned out similar to something else or one of them is more defined than another*”. As a consequence, these candidate domains were consolidated into a single overarching health-related concept, named ‘fear’ (Fig. 1). The plain language description was expanded to include all concepts (including fear for health, now and in the future and fear of it getting worse).

Similar comments made by Public Research Partners and Lay Reviewers also prompted the consolidation of the domain ‘bothered’ with ‘annoyance’. For example, VC said “*Do we need all of these?... Is annoying disruptive? As much as disruptive isn’t it?...Disturbing.*” Three Lay Reviewers said “*What's the difference between being 'annoyed' and being 'bothered'? In fact, what, specifically, does 'bothered' mean?*” and “*Questions 29 [tinnitus annoyance] and 30 [bothered] feel very similar to me, and difficult to distinguish between them clearly.*” and “*What's the difference between this, 'annoyed' and 'bothered' - and 'irritable' in the later aspect?*”. The plain language description for ‘annoyance’ was expanded to include elements of bothered (i.e. knowing tinnitus is there and finding it a nuisance).

Lay Reviewers also considered ‘frustration’ to share important conceptual similarities with ‘upset’ and as a consequence, ‘frustration’ was consolidated with ‘upset’. Their feedback also prompted us to change the plain language description for ‘upset’ to more closely match the definition given in the Oxford English Dictionary [Oxford English Dictionary, 2017]. The Study Management Team was satisfied that this new description also encapsulated aspects of the associated concept ‘frustration’.

#### Consolidating outcome domains that define how the domain might be measured

A number of outcome domains were judged to relate more directly to how an outcome domain could be measured. For example, an ‘active task to distract or cope with tinnitus’ and ‘purposefully protecting or reducing the chance of potential problems’ were both considered different ways to measure the same health construct ‘coping’. This recommendation from the Public Research Partners resulted in ten outcome domains being consolidated in the final version of the long list under existing outcome domains ‘tinnitus loudness’, ‘tinnitus quality’, ‘coping’, and the category ‘body structures and functions’.

### Modifying outcome domain names

Discussion with the Public Research Partners’ highlighted ambiguities in meaning and awkwardness of repeated language for some outcome domain names, particularly in the ‘health-related quality of life’ category (see Table 1).

**Table 1:** Illustrative examples of modifications to the outcome domain names for domains in the ‘health-related quality of life’ category

Original outcome domain name	Modified outcome domain name
Interference on social activities	Impact on social life
Interference on work activities	Impact on work
Interference on activities	Impact on individual activities
Interference on relationships	Impact on relationships

In one example, they spoke about how group-based activities present very different challenges compared with individual activities. As a result, the domain name was modified to emphasise the individual nature of the activities in question (Table 1). A transcript of part of the discussion about the outcome domain named ‘interference on activities’ is reported below:

VC: “*An activity sounds physical doesn’t it, rather than…*”

BT: “*So a social*”

VC: “*Gathering*”

BT: “*Gathering, because that could be anywhere*”

VC: “*That could be restaurant, pub, party*”

VC: “*If you’re asking people for an honest reaction to how much it interferes with their activities, there are two very different things. One is on a one-to-one erm social activity with somebody and the other is a group gathering or activity with people…It’s much easier to control the one-to-one…*”

In a second example, there was discussion about the term ‘interference’ and its repeated usage which led to its substitution with the term ‘impact’ (Table 1):

VC: “*Isn’t it, rather than interfere…Interfere is quite a negative word isn’t it?*”

KF: “*Well yeh, we try to use impact over interfere because impact can be… its more posi…, so you can have a positive impact and a negative impact…*”

VC: “*Yeh*”

KF: “*…Whereas interfere just comes across as...*”

VC: “*Very negative*”

KF: “*It’s negatively interfering with something*”

HS: “*Effecting or impact would be quite neutral*”

### Modifying the plain language descriptions

Table 2 reports three illustrative examples of how input from patients informed changes in our descriptions to make them more understandable and relevant. Lay Reviewer comments were especially informative for understanding how other people interpreted the outcome domains according to the plain language descriptions that had been co-created by the Public Research partners and the Study Management Team. For a number of domains, Lay Reviewers’ suggestions prompted us to add more examples to widen the range of possible experiences or scenarios. Inviting feedback from a wider group, with a more diverse range of lived experiences with tinnitus, enabled us to enhance the personal relevance of our descriptions.

**Table 2:** Three illustrative examples of how input from patients informed changes in our descriptions to make them more understandable and relevant to their experience of tinnitus

Outcome domain name	Initial description	Final description	Explanation for the change
Confusion	Feeling uncertain or unclear	Being unable to think clearly, either in general or specifically associated with your tinnitus	Patients told us we needed to clarify what was causing the confusion. We also had to be careful to distinguish from another complaint called “worry/concerns” which also had the word “uncertain” in its definition.
Tinnitus pitch	Whether the tinnitus has a high note or a low note	Whether your tinnitus has a note-like quality, for example high pitch like whistling or low pitch like humming	Patients told us we needed to consider different values of pitch so we broadened the description with examples. Changed from “the tinnitus” to “your tinnitus” to make it more personally directed.
Upset	To be made unhappy by tinnitus	Feeling unhappy or disappointed because of your tinnitus	A patient recommended using the Oxford English Dictionary definition. We also substituted “feeling” instead of “to be made”, and added “because of your tinnitus” to make it more personally directed.

### Potential impact on the round 1 Delphi survey

Although it is not possible to directly attribute study performance to the public involvement described above, the online Delphi survey was successful in recruiting and retaining participants. Evidence indicates that participants from a diverse range of backgrounds had positive experiences of taking part.

#### High participant recruitment

Recruitment exceeded expectations. Although recruitment was open for only a 14-week period (April–July 2017), it surpassed the target of 420 experts [5], with 719 participants consenting and 670 completing round 1 of the Delphi surveys. The COMIT’ID Delphi survey appealed equally to patients (*n* = 358) as well as to professionals (*n* = 312). This contrasts with a number of other COS studies which have recruited markedly lower numbers of patients compared to professionals (e.g. [14, 23, 30, 31]).

#### Positive participatory experience

Accessibility of the Delphi survey is illustrated by the following selection of feedback quotes given by patients:

SOUND00016: “*It took a little while for me to understand the concept but once I got it; it was fine.*”

SOUND00078: “*Very good questions. I did not know that what I have been thinking so long was even real; or just real for me.*”

TALKC00200: “*Straight forward to complete.*”

DRUGC00051: “*Easy to understand and follow. Ready for the next round.*”

Following the GRIPP2-SF checklist [27], consideration was also given to any negative participatory experiences. These were relatively few, but some participants expressed general difficulties in understanding some of the outcome domain concepts, with comments including the following:

SOUND00064: “*I'm not sure I understand the subtle distinction between some categories – e.g. several mention behaviour; several mention coping and dealing with tinnitus. I've tried my best to separate them.*”

SOUND00147: “*As a member of the public with tinnitus a number of the questions were difficult to understand and/or answer; either because the language used was too technical or because it was hard to see how it related to either tinnitus or the treatment thereof.*”

TALKC00188: “*A little difficult to understand what I'm being asked. Questions need to be more clear.*”

Post-hoc analysis explored whether non-native English language might be a barrier to participation, but ruled out this possibility for the following reasons. First, 233 of the 719 participants declared that they did not speak English as a native language (47 of these were patients). Second, although there was an option to leave a comment against each domain, we received very few comments (*n* = 10) that were concerned with a lack of understanding individual domains. Only four of those were from people who did not have English as their first language. For example, one French healthcare professional (SOUND00190) said “*For a hearing-impaired person this question may be ambiguous. One of his major concern is to understand speech when noise is present in his surrounding. Do what does “confusion” refers to? Does it relate to the understanding of the conversation or clear thoughts”.* A full list of feedback comments gathered during the first round of the Delphi survey can be found in Additional file 1: Table S1.

#### High retention

Furthermore, there is no reason to believe that the length of the long list was off putting to patient participants. First, attrition was very low. Of the 670 completing round 1, 586 also completed round 2, giving a 87% overall retention rate. Retention was similar for patients (305/358, 85%) as it was for professionals (281/312, 90%). Second, in round 1 only one negative comment was received from a patient about the length of the survey (SOUND00294 said “*Too long... I just want some help please*”).

High retention of patients in the COMIT’ID Delphi survey contrasts with a number of other COS studies in which patients, more so than professionals, have withdrawn across rounds 1 and 2. In one extreme example, Al Wattar [30] reported 0% retention for patients (0/24) compared with 68% for professionals (35/51). In another example, the COS study [14] achieved a reasonable 78% retention for patients (25/32), but not as good as the 91% for professionals (149/163).

## Discussion

Patient involvement was an integral and transformative step of the study design process. Through the inclusion of Public Research Partners with lived experience of tinnitus and a PPI/E Manager as three out of the nine members of the Research Steering Group, the Study Management Team co-produced a final long list comprising 66 outcome domains describing distinct tinnitus-related complaints, with plain language domain names and concise but understandable descriptions for each.

To the best of our knowledge, no previous COS studies seem to report detailed public involvement methods for optimising the long list of candidate outcome domains. It is interesting to note that the recent COMET handbook v1 [Section 2.9.1.3, 9] discusses the value of qualitative research findings from patient interviews and focus groups for ensuring that the outcome domain names and explanations are understandable to patients, but does not specifically mention the role of patient involvement in this process. In this way, the procedures and insights described in this article not only made valuable contributions to the successful conduct of our Delphi survey, but also contribute to the broader methods literature by demonstrating how patient involvement can enhance the accessibility of the consensus processes.

This Discussion highlights seven general methodological features which the Research Steering Group feels made a substantial difference and which fit with general principles of good patient involvement. These recommendations for effective patient involvement in refining a long list of candidate outcome domains in a modified Delphi survey are briefly explained below.

1. **Planning:** *Plan what PPI steps are likely to have most beneficial impact on the Core Outcome Set project and incorporate them into the ethical approval process.*
  Although ethical approval is not mandatory for public involvement, setting out these steps in the study protocol that was approved by the Research Ethics Committee was a valuable element of our project planning (see also [32]). By specifying the roles of the Public Research Partners and other healthcare users, it was possible to fully inform lay members what was expected from them during the recruitment process. While VC was an experienced Public Research Partner with whom the Study Management Team had worked previously, BT was recruited to this role after responding to an advertisement and completing an informal telephone interview with HS. This approach would not have been possible without prior planning. Second, by clearly defining the public involvement roles it was possible to budget appropriately for the associated costs in the project. An additional grant was subsequently obtained from the British Tinnitus Association specifically to support the public involvement component of the COS development, and this application was led by the PPI/E Manager and with one of the Public Research partners (VC) as a co-applicant. Agreeing the roles of the lay members and appropriate budgeting are two recognised principles of good practice for successful healthcare user involvement in National Health Service research [33]. Moreover, involving the Public Research Partners in the grant application and as members of the Research Steering Group were opportunities to formally acknowledge the importance of their role.
2. **Seeking relevant input:** *Appoint a small number of Public Research Partners with lived experience of the condition of interest, an eagerness to express their opinions, and whom can provide continuity over a number of face-to-face workshops. A PPI/E Manager can be helpful in providing personal support and mentorship.*
  The two Public Research Partners shared a willingness to express their opinions and a sense of humour. These were two positive personal qualities that made the public involvement process enjoyable, not only for the individuals involved but also for the Study Management Team. It was helpful that the Public Research Partners lived locally and were retired because this made it possible for them to provide continuity over a number of face-to-face workshops. Personal access to internet and email were also beneficial for sharing documentation before and after the workshops, and as members of the Research Steering Group. The involvement of the PPI/E Manager was also useful in providing personal support and mentorship, whenever required. It can be reassuring to have a professional member of the Research Steering Group who has not been involved in designing the study, providing an impartial contact for healthcare users and a balance of perspectives. Again, provision of lay support meets one of the principles of good public involvement [33].
3. **Being open-minded:** *Remain open minded, flexible, and curious about the lay perspective.*
  In this study, public involvement was far from tokenistic. The Study Management Team remained open minded, flexible and curious about the lay perspective. They were not simply expecting the Public Research Partners to confirm the initial long list of candidate outcome domains. In fact, by respecting their knowledge and experience, the Study Management Team welcomed the challenge at the first workshop to reconsider the length of the preliminary long list. The final long list of candidate outcome domains was directly shaped in response to this challenge. It was bespoke according to the needs of the study as seen through the eyes of our Public Research Partners and Lay Reviewers.
4. **Careful reviewing:** *Review the preliminary long list of candidate outcome domains in a face-to-face workshop(s), focusing discussion on the names, underlying theoretical constructs and plain language descriptions of each one.*
  The face-to-face workshop format worked well in enabling facilitated discussions about the underlying theoretical constructs and plain language descriptions. To maintain a balance of membership, a workshop was convened with an equal number of researchers to Public Research Partners. Having a PPI/E Manager participate in the workshop was helpful in providing support to the Public Research Partners and prompting the researchers when technical terms required further explanation or when clarification was needed. A single workshop was insufficient. After 3½ hours of discussion, there were still domains on the preliminary long list which had not been considered. Participation was an intense (and tiring) experience and so a second workshop was planned to complete the work. In hindsight, the Study Management Team had perhaps been too ambitious to expect to complete discussion of all 124 domains in one meeting. This lesson has been taken on board when planning the agenda for the final face-to-face consensus meeting after the Delphi survey is completed. Meaningful discussion on all 66 outcome domains in the final long list is unlikely to be achieved in a one-day meeting.
5. **Note taking:** *Take notes and audio record the workshop discussions for later reference.*
  Handwritten notes were important, but an audio recording of the workshop discussions was invaluable for later reference to jog the memory of the researchers. Not all decisions about modifying the long list were made in the workshops themselves. The notes and audio recordings were therefore important in assisting the Study Management Team to reflect on the content of the discussions and to make informed decisions without any time pressures and also to reflect again on the discussion to make sure that none of the researchers were ‘putting words in the mouth’ of the Public Research Partners. The audio recording was particularly helpful to the member of the Study Management Team (DAH) who was not present in the workshops. It gave a retrospective yet accurate record of who said what. The Study Management Team also referred to the audio recording when preparing this research report.
6. **Reflecting:** *Reflect on the discussion and recommendations as a Study Management Team to create an interim long list.*
  Taking time outside the two Public Research Partner workshops gave an important opportunity for the Study Management Team to reflect on the discussion and recommendations without feeling any undue time pressures. Members of the Study Management Team who did attend the workshop also found it helpful to go over the main points of the conversation with their fellow member who had not been present in the workshop, so giving a new perspective.
7. **Engaging wider feedback:** *Identify and engage a diverse group to review the interim list of outcome domain names and plain language descriptions, thus creating a final long list.*
  Review of the interim long list by a reasonably broad mix of patients and clinical experts increased our confidence in the conceptual uniqueness of each domain and personal relevance of each plain language description. The experience in round 1 was that none of the feedback challenged our grouping or definitions of the candidate outcome domains. From this the conclusion is that reducing the overlap in the constructs corresponding to each outcome domain at the preparatory stage reduced the likelihood of Delphi participants recommending changes to the long list. Rather than querying the validity of the outcome domains themselves, participants have focused on scoring importance. In at least one preceding COS study, participant feedback was found to challenge investigators to exclude some overarching domains in between the Delphi rounds [31]. It is uncertain whether this earlier study incorporated patient involvement at the preparatory stage.

The study findings suggested that non-native English was not a barrier to participation in the English language Delphi survey. Nevertheless, including patient involvement with non-native English language speakers could have brought additional benefits by enabling a more representative group of people to comment on their understanding of the outcome domain names and descriptions. This limitation could have been overcome by engaging the European professional members of the Research Steering Group to lead some of the patient involvement activities in their own countries, either in the form of workshops or an online survey. Enlisting patient involvement from those with a range of English language abilities is just one aspect of diversity. Involving people with a range of ages, ethnicity and socio-economic status are other aspects to consider in future work. The Study Management Team did not act on the small number of negative participatory experiences that were received in the round 1 feedback, due to resource limitations and time pressures for opening round 2. However, future work could consider a role for patient involvement here, especially where comments pertain to the understanding of specific outcome domain names and descriptions.

The time spent developing a high-quality long list of candidate outcome domains as preparation before the Delphi process is anticipated to bring later benefits in the face-to-face consensus meetings. Several COS study reports show how considerable discussion time during the consensus meeting has been taken up with queries about consolidating outcome domains into larger categories, excluding some domains on the basis that they are more relevant to how the domain is measured or that they are specific to one form of intervention, or sometimes even redefining the outcome domains themselves. In one example, during a COS consensus meeting for otitis media with effusion in children with cleft palate, participants agreed to combine the outcome domain ‘consonant production (cleft-related speech patterns)’ with ‘consonant production’, and furthermore that both of these should directly inform subsequent decisions of how to measure ‘speech development’ [13]. In another example, during a COS consensus meeting for psoriatic arthritis, participants agreed to redefine the outcome domain ‘emotional well-being’ (feeling good about oneself) so that it additionally captured the outcome domains: ‘anxiety’, ‘depressive mood’, ‘embarrassment’, ‘frustration’, ‘self-worth’, and ‘stress’ [34].

For the COMIT’ID study, the Study Management Team has pre-empted many of these discussions in the preparatory phase, leaving greater opportunity for the consensus meeting to focus on prioritising what should be in the core set.

## Conclusions

The end result of the public involvement process has been a rich insight into the healthcare user perspective for the design of a long list of candidate outcome domains for a modified Delphi survey. This article describes procedures and offers methodological guidance for working effectively with patients to develop a mutual understanding of (often complex) healthcare concepts, and to co-produce the final long list with a set of plain language descriptions. Although it is not possible to directly attribute study performance to public involvement, the online Delphi survey has been highly successful in terms of recruitment, retention and accessibility. This experience confirms the wide-ranging benefits of public involvement in reviewing the long list before an online Delphi survey is launched. Procedures recommended in this article go beyond core outcome set development because they embody general standards for good patient involvement that can be transferred into other research contexts.

## Additional file

Additional file 1: Table S1. A full list of the general feedback comments received by participants completing round 1 of the online Delphi survey. (DOCX 26 kb)

## Abbreviations

COMIT’ID: Core Outcome Measures in Tinnitus International Delphi
COS: Core outcome set
COST: Cooperation in Science and Technology
PPI/E: Patient and Public Involvement and Engagement
TINNET: European TINnitus NETwork
