# Supplementary material for: Defining and evaluating novel procedures for involving patients in Core Outcome Set research: creating a meaningful long list of candidate outcome domains
Source: Res Involv Engagem. 2018 Mar 2;4:8. doi: 10.1186/s40900-018-0091-5 (PMC5833049; doi:10.1186/s40900-018-0091-5)
Supplement: Supplementary file 1 — A full list of the general feedback comments received by participants completing round 1 of the online Delphi survey. (DOCX 26 kb) [file 40900_2018_91_MOESM1_ESM.docx]

**Additional file 1: Table S1** A full list of the general feedback comments received by participants completing round 1 of the online Delphi survey.

| **Outcome domain** | **All round 1 comments concerning lack of understanding** |
| --- | --- |
| Active myofascial trigger points | English-language speaker  *“Question isn't clear.”* (Patient TALKC00085). |
| Behaviour | English-language speakers   - *“This outcome is a bit vague and not sure how to score.”* (Researcher SOUND00321). - *“Did not really understand what was needed here as an individual* (Patient SOUND00250). - *“This outcome is not clear."* (Patient TALKC00031).   Non-native English-language speaker   - *“Please specify; what types of behaviour? social; fear?”* (Commercial representative DRUGC00161). |
| Confusion | Non-native English-language speakers   - **“***I don't understand the question.”* (Patient TALKC0007). - **“***For a hearing-impaired person this question may be ambiguous. One of his major concern is to understand speech when noise is present in his surrounding. Do what does "confusion" refers to? Does it relate to the understanding of the conversation or "clear thoughts".”* (Healthcare professional SOUND00190). |
| Distress from bodily sensations | English-language speaker   - *“I don't know if T can be regarded as a physical feeling. If it cannot; then I don't know what this question means.”* - (Patient SOUND00083). |
| Feeling tired | Non-native English-language speaker   - *“I do not understand this question or any of the ones that follow on this page!!!”* (Patient TALKC00105). |
| Need for knowledge | English-language speaker   - *“I don't understand how this is measurable as an outcome. Is wanting to gain knowledge regarded as a good thing or a bad thing?”* (Patient SOUND00083). |
